# Unique case of profound iatrogenic hypercalcemia in a patient with recent orthopedic prosthetic infection 

**DOI:** 10.5414/CNCS110179

**Published:** 2020-11-19

**Authors:** Yela Jung, Kyaw Moe, Everado Arias Torres, Kamyar Kalantar-Zadeh, Ramy M. Hanna

**Affiliations:** 1 Department of Medicine, University of California Irvine,; 2 Department of Medicine, Long Beach Memorial Medical Center, Department of Medicine Division of Nephrology,; 3 Department of Medicine, and; 4 Department of Medicine, Division of Nephrology and Hypertension and Kidney Transplantation, University of California, Irvine, CA, USA

**Keywords:** Stimulan beads, hypercalcemia, symptomatic hypercalcemia, bisphosphonates, normal saline

## Abstract

Hypercalcemia is a common electrolyte disorder and is typically caused by parathyroid-dependent and parathyroid-independent causes. The most common parathyroid-independent causes include malignancy, granulomatous diseases, over-supplementation with calcium, and hypervitaminosis D. We present an unusual case of a woman who had Stimulan implanted after an artificial knee joint infection. When a washout was done, the patient’s serum calcium started rising, peaking at an astounding 21.2 mg/dL (normal range 8.4 – 10.2 mg/dL) with acute kidney injury. After aggressive hydration and treatment with furosemide, bisphosphonates, and calcitonin, the serum calcium dropped to 10.1 mg/dL. A full hypercalcemia workup did not reveal an alternate cause. On further investigation, it was found that Stimulan is calcium based, and the agitation of these beads during washout was hypothesized to result in the observed profound hypercalcemia.

## Introduction 

Hypercalcemia is a common medical finding with a broad differential diagnosis. The parathyroid-related causes are typically due to primary hyperparathyroidism or tertiary hyperparathyroidism [1]. Non-parathyroid-related processes include calcium overdose, hypervitaminosis D, inborn errors of calcium metabolism, granulomatous disease (fungal disease, sarcoidosis), malignancy (via parathyroid hormone-related peptide), and other causes [2]. 

The general treatment involves hydration and bisphosphonates [3]. Newer options include receptor activator of nuclear factor κ-Β ligand (RANKL) monoclonal antibodies like denosumab given at higher doses [4]. We present an unusual case of hypercalcemia in the setting of antibiotic-coated Stimulan beads, agitated during a washout of an infected knee transplant, that resulted in profound hypercalcemia. 

## Case description 

The patient was a 58-year-old woman with past medical history of hypothyroidism that was stably controlled on a stable dose of levothyroxine, hypertension, osteoarthritis, chronic pain on methadone, and bipolar disorder who presented with fatigue, nausea, vomiting, confusion, and generalized weakness for 7 days. She was hospitalized a week prior to developing symptoms for an infected right knee arthroplasty with subsequent removal of hardware and placement of antibiotic spacer and beads ~ 1 week prior and was discharged on intravenous (IV) vancomycin via peripherally inserted central catheter (PICC line). She had been doing well until 2 days after discharge and had then progressively become more lethargic. 

In the Emergency Department, the patient was confused with tenderness to light palpation of epigastrium. She was hypertensive to 177/65 with heart rate of 57 and normoglycemic with point-of-care glucose of 105 mg/dL. She was found to have severe hypercalcemia of up to 21.2 mg/dL with ionized calcium level of < 2.5 mmol/L and albumin 3.0 gm/dL, acute renal failure with creatinine of 1.6 mg/dL, and pancreatitis with lipase of 3,430 U/L. Other lab results were notable for low parathyroid hormone (PTH) of 14.9 pg/mL, 1,25-vitamin D of < 5 pg/mL, and 25-OH vitamin D of 9 ng/mL. The suppressed parathyroid hormone level made secondary hyperparathyroidism less likely, despite the presence of vitamin D deficiency in this case. 

Parathyroid hormone-related peptide (PTHrP) level was within normal limits at < 2.5 pmol/L. Chest X-ray performed on this admission also was normal without any mass. Serum protein electrophoresis showed hypoproteinemia with hypoalbuminemia without a monoclonal protein (M-protein) spike. Urine protein electrophoresis showed protein of 8 mg/dL with albumin as predominant protein. Both anti-nuclear antibody (ANA) and complement levels (C3, C4) were unremarkable. According to these lab values, primary hyperparathyroidism, hypervitaminosis D, paraneoplastic syndrome, granulomatous disease, and multiple myeloma were ruled out. She was given methylprednisolone 4 mg IV once, furosemide 40 mg IV every 6 hours (given development of hypervolemia as per guidelines) [5], normal saline at 250 cc/hour, pamidronate 90 mg in normal saline 500 mL Intravenous Piggy Back (IVPB) once, and calcitonin injection 285 units (for 48 hours). Due to her acute renal failure, her antibiotic regimen was also changed from vancomycin to daptomycin 6 mg/kg every 2 days. 

**Figure 1.**
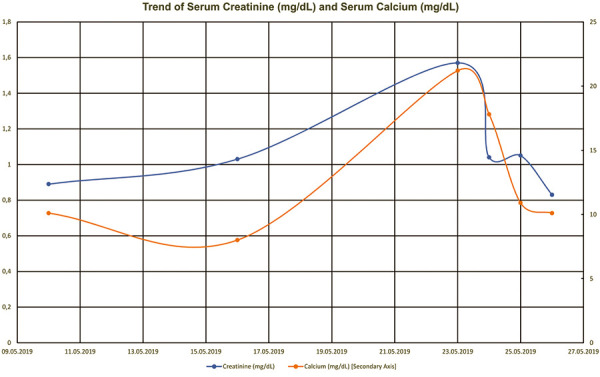
Trend of serum calcium and creatinine after washout of infected joint with Stimulan beads. Creatinine (Cr) mg/dL, Calcium (Ca^2+^) mg/dL.

Prior to this episode, the patient had no prior history of renal dysfunction (baseline creatinine 0.8 – 0.9 mg/dL and estimated glomerular filtration rate > 60 mL/min/1.73m^2^), malignancy, or recent change in medication that could be the culprit of her calcium level. Her pre-operative medication included biotin 10,000 μg) daily, duloxetine 60 mg daily, quetiapine 25 mg every night, amlodipine 10 mg daily, carisoprodol 350 mg 3 times a day, diclofenac sodium 50 mg 3 times a day, fenofibrate 134 mg daily, gabapentin 800 mg 3 times a day, lamotrigine 200 mg every morning and 250 mg every night, levothyroxine 88 μg) every day, methadone 10 mg every 4 hours if needed, methocarbamol 750 mg 3 times a day, multivitamin 1 tablet daily, orphenadrine 100 mg twice a day, oxycodone 15 mg every 4 hours if needed, tizanidine 4 mg every 6 hours, and triamterene-hydrochlorothiazide 37.5 – 25 mg capsule daily. Post-operatively, she was started on aspirin 325 mg daily, intravenous cefazolin 2 g every 8 hours, dilaudid injection 0.5 mg every 3 hours as needed, and morphine injection 2 – 4 mg every 2 hours as needed. She had a history of hypothyroidism but was well-controlled on the same dose of levothyroxine. Also, her bipolar disorder was controlled with duloxetine, quetiapine, and lamotrigine without the need for lithium. Within the next 3 days, she markedly improved as her calcium level came down with the above treatments. On discharge, her calcium was at 10.1 mg/dL with creatinine back to baseline (Figure 1). Her most recent calcium level after discharge was 8.8 mg/dL, and subsequent urinalysis was unremarkable. She had no further event of hypercalcemia or acute renal failure after this hospitalization. 

The acute time course and rapid recovery indicated an iatrogenic cause of her symptomatic hypercalcemia. In examining her hospitalization to treat her infected right knee arthroplasty, it was found that Stimulan was used, which is composed of calcium sulfate. When her infected joint was washed out, antibiotic-loaded acrylic cement, 9 L of triple antibiotic sterile saline solution and chlorhexidine lavage, and 50 mL of Stimulan containing 1 g of vancomycin and 240 mg of tobramycin per 10 mL were used to attempt to clean the knee replacement to salvage artificial joint. Cortisol levels were not measured, but opioid-induced hypocortisolemia may have played some role with regard to lethargy. It is unlikely, however, that they directly contributed to hypercalcemia since patient was not hypotensive or hypoglycemic and without any other signs of hypocortisolemia. 

## Discussion 

Total knee replacement is one of the most common orthopedic procedures that has been used to significantly improve patients’ lives. The most common complication involving any hardware is infection. Surgery is the gold standard treatment for these infections, including multiple debridement of affected tissue, irrigation with antibiotic-containing solution, and insertion of antibiotic eluting dissolvable beads and antibiotic loaded cement. 

Stimulan beads are dissolvable calcium sulfate that substitutes as a bone graft and helps with sustained release of antibiotics over time [4, 5, 6, 7, 8, 9, 10, 11]. These are often used due to their 100% antibiotic release and higher sustained concentrations over several weeks [11]. However, there have been a few case reports that describes patients with symptomatic hypercalcemia, requiring treatment after receiving Stimulan implantations [6]. 

A case series involving 755 patients found an associated risk of transient hypercalcemia and use of Stimulan beads [7]. 41 patients (5.4%) had transient hypercalcemia with a mean calcium level of 11.7 mg/dL (10.8 – 14.9 mg/dL), and 2 were symptomatic, needing treatment with fluids and an intravenous dose of bisphosphonate [7]. Calcium levels in these patients increased and resolved back to normal within 5 days post-operatively [7]. Furthermore, there was a higher number of patients with hypercalcemia in those implanted with larger volume of beads. The highest bead volume in the studied patients with hypercalcemia was 50 mL, concurrent with the volume used in our patient [7]. A case report describes a woman with severe hypercalcemia of up to 12.2 mg/dL 2 days after placement of calcium sulfate beads for a left hip infection [8]. She was treated with aggressive IV fluids, multiple doses of calcitonin, and eventually 2 sessions of hemodialysis due to worsening renal failure and volume overload [8]. Another case report describes a woman who developed severe hypercalcemia of up to 16.1 mg/dL 6 days after right knee joint arthroscopy with calcium sulfate bead placement [9]. She was also treated with aggressive IV fluids and 1 dose of calcitonin [8]. Similar to our patient, neither women described in the above case reports had any further event of hypercalcemia or renal failure. 

Currently, post-operative monitoring for any orthopedic replacement is focused on pain, infection, and function of joints. Labs ordered at follow-up orthopedic appointments may include a complete blood count with differential, erythrocyte sedimentation rate, and C-reactive protein to rule out periprosthetic infections [12]. At this time, there is no clear guideline for post-operative monitoring following Stimulan insertion. 

According to the pharmaceutical producer, many studies have been done to show the performance for bone healing and against infections, biofilms, and drainage [11]. The United States Food and Drug Administration has approved its use for mixing with vancomycin, gentamicin, and tobramycin [11]. However, there was an adverse reaction report in 2005 regarding hypercalcemia after placement of vancomycin-infused Stimulan in a patient undergoing left hip arthroplasty [12, 13]. Further studies need to be done to observe whether these different antibiotics may influence the release of calcium from Stimulan. 

Despite Stimulan’s valuable use in treatment of orthopedic infections [11], caution must be taken for possible complication of hypercalcemia. Especially in patients with elevated risk factors, such as renal failure, calcium levels must be monitored before and after surgery. Depending on the severity of hypercalcemia, different treatment methods can be used. Patients with < 12 mg/dL (3.0 mmol/L) are often asymptomatic and therefore do not require urgent correction [4, 14]. Patients with 12 – 14 mg/dL (> 3.5 mmol/L) may not require treatment if the level has increased slowly over time, however, if it is an acute increase and/or the patient is symptomatic, they need to be treated [4, 14]. Per current guideline, initial treatment is rehydration with up to 4 – 6 L of intravenous 0.9% saline boluses within 24 hours [14]. If further treatment is required, intravenous bisphosphonates can be used afterwards [3, 14]. Other second-line treatments include glucocorticoids, calcitonin, denosumab, and parathyroidectomy [4, 14]. This rare case of iatrogenic hypercalcemia in a patient with recent hardware infection shows the possible negative effect of Stimulan beads. An interesting research question is whether Stimulan beads interact with different antibiotics, possibly affecting calcium release. There is no clear data on this, but it is known that different antibiotics can alter bone cement hardening after Stimulan bead insertion into the joint. This is a valuable topic for future research regarding which antibiotic/bead combinations maybe carry the highest risk for transient hypercalcemia [15]. 

## Ethics approval and consent to participate 

Not applicable. 

This research work does not contain human subject research material. 

## Ethical permission/consent for publication 

Not applicable. 

## Availability of data and materials 

Not applicable, no data. 

## Funding 

Kamyar Kalantar-Zadeh is supported by the National Institutes of Health-National Institute of Diabetes, Digestive and Kidney Disease (NIH-NIDDK) grant K24-DK091419 as well as philanthropist grants from Mr. Harold Simmons, Mr. Louis Chang, Dr. Joseph Lee, and AVEO. 

## Conflict of interest 

None.
